# Druggability Analysis of Protein Targets for Drug Discovery to Combat *Listeria monocytogenes*

**DOI:** 10.3390/microorganisms12061073

**Published:** 2024-05-25

**Authors:** Robert Hanes, Yanhong Liu, Zuyi Huang

**Affiliations:** 1 Department of Chemical and Biological Engineering, Villanova University, Villanova, PA 19085, USA; rhanes01@villanova.edu; 2 Eastern Regional Research Center, U.S. Department of Agriculture, Wyndmoor, PA 19038, USA

**Keywords:** druggability, *Listeria monocytogenes*, binding pockets, logistic regression model, docking

## Abstract

Extensive research has been conducted to identify key proteins governing stress responses, virulence, and antimicrobial resistance, as well as to elucidate their interactions within *Listeria monocytogenes*. While these proteins hold promise as potential targets for novel strategies to control *L. monocytogenes*, given their critical roles in regulating the pathogen’s metabolism, additional analysis is needed to further assess their druggability—the chance of being effectively bound by small-molecule inhibitors. In this work, 535 binding pockets of 46 protein targets for known drugs (mainly antimicrobials) were first analyzed to extract 13 structural features (e.g., hydrophobicity) in a ligand–protein docking platform called Molsoft ICM Pro. The extracted features were used as inputs to develop a logistic regression model to assess the druggability of protein binding pockets, with a value of one if ligands can bind to the protein pocket. The developed druggability model was then used to evaluate 23 key proteins from *L. monocytogenes* that have been identified in the literature. The following proteins are predicted to be high-potential druggable targets: GroEL, FliH/FliI complex, FliG, FlhB, FlgL, FlgK, InlA, MogR, and PrfA. These findings serve as an initial point for future research to identify specific compounds that can inhibit druggable target proteins and to design experimental work to confirm their effectiveness as drug targets.

## 1. Introduction

It is estimated by the Centers for Disease Control and Prevention (CDC) that about 48 million cases of foodborne illnesses occur in the United States every year. Among them are approximately 1600 listeriosis infections that cause about 260 deaths, a 94% hospitalization rate, and a 16% mortality rate [1,2]. The fatality rate reaches as high as 30% for people with weakened immunity (e.g., the elderly and children) and pregnant women [3]. Listeriosis is caused by the foodborne pathogen *Listeria monocytogenes*, which is the third leading cause of death among pathogens that cause foodborne illnesses [4]. *L. monocytogenes*, as a facultative intracellular pathogen, can endure various stress conditions [5]. It is reported as a prevalent pathogen across various countries, such as the United Kingdom, the United States, Canada, Australia, and Mexico [6]. *L. monocytogenes* is commonly found in environmental settings and is carried by animals, with humans primarily contacting the bacteria through contaminated foods and surfaces [7]. Consequently, *L. monocytogenes* poses significant concerns for the food industry [8]. The primary treatment for listeriosis typically involves ampicillin, administered either alone or in combination with gentamicin [9]. Although the presence of ampicillin-resistance genes in *L. monocytogenes* has not shown an increasing trend [10], the ongoing risk of resistance persists due to lateral gene transfer in bacteria [11]. Identifying novel methods to control *L. monocytogenes* is thus needed to ensure the continued availability of effective treatments for infected patients.

*L. monocytogenes* survives in the environment via its ability to react to external stresses by producing key proteins that facilitate adaptive reactions [12]. These stress response factors enable *L. monocytogenes* to survive in conditions including low moisture, high salt concentration, and refrigeration [13]. Previous studies have identified important stress response factors; for example, *sigB* plays a critical role in osmotic and cold stress responses [14]. Once the pathogen survives, it expresses proteins that enable it to invade host cells, evade host immune defenses, and initiate infection [15]. When treated with antimicrobials, the pathogen may express antimicrobial-resistance genes to defeat drugs designed to eliminate it [16]. Identifying targets that disrupt the stress response, antibiotic resistance, and virulence provides multiple vectors to control *L. monocytogenes*. For example, these targets could enhance food preservation techniques to diminish the occurrence of *L. monocytogenes* in the food chain, diminish virulence to alleviate the severity of infections in individuals exposed to the pathogen, and counteract the emergence of antibiotic resistance by discovering novel or synergistic compounds to maintain the efficacy of existing therapies. Enhancements in any of these areas can yield favorable effects in the treatment of *L. monocytogenes*. For instance, anti-virulence medications targeting specific virulence factors could serve as viable alternatives to traditional antibiotic therapies [17].

However, the development of new treatments presents a significant challenge. From 2008 to 2010, 51% of Phase II drug failures were due to a lack of efficacy [18]. Bayer funded a study in which the properties of a good drug target were defined [18]. Two key properties of effective drug targets have been defined as follows: (1) the target should play a functional role in the disease process, and (2) the target should be druggable [18]. Druggability means that a functional part of the target can be bound by a drug, such as a chemical compound [18]. In the case of proteins, drugs are essentially ligands that attach to the proteins and modify their functionality, and the protein structures can be analyzed for pockets in which ligands can bind [19]. In this work, an approach is developed to evaluate protein structures to identify pockets and to assess the likelihood of ligand binding as a measure of druggability to evaluate protein targets for *L. monocytogenes*.

While ongoing research is being conducted to discover novel drugs or inhibitors targeting *L. monocytogenes* [13,20], more work is needed to evaluate potential targets for new drugs and assess their druggability. Prior studies have identified key genes and proteins involved in the stress response, virulence, and antibiotic resistance of *L. monocytogenes* [10] that could serve as targets for new methods of control. However, little work has been conducted to further evaluate the druggability of these targets. This work aims to fill this gap by developing a druggability-evaluation model based on the structural characteristics of binding pockets in proteins inhibited by known inhibitors and FDA-approved drugs (mainly antimicrobials). Subsequently, the developed model will be utilized to assess potential protein targets of *L. monocytogenes* highlighted in the existing literature. 

## 2. Materials and Methods

**Figure 1:**
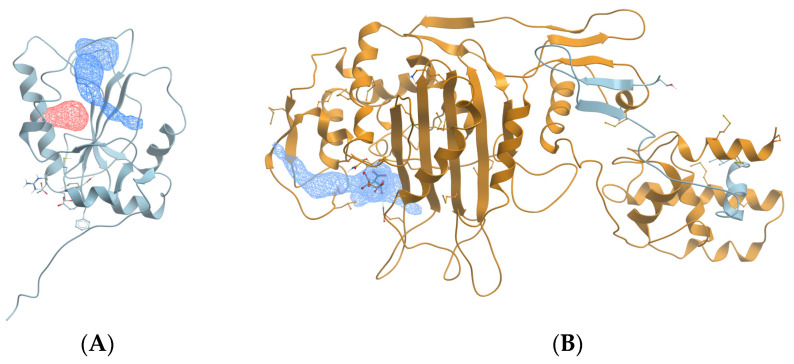
(**A**) Example protein structure (PDB 1C25) showing 2 pockets (blue mesh and red mesh) without ligands; (**B**) example protein structure (PDB 3ZG9) showing 1 pocket (blue mesh) with ligand.

In this work, an approach was developed to assess the druggability of proteins by analyzing their binding pocket structures in *L. monocytogenes*. A protein is considered druggable if a drug molecule can bind to one of its binding pockets and impact its typical function [21]. Molsoft ICM-Pro software (Sorrento Valley, CA, USA) is one of the best platforms for identifying pockets that ligands can dock to and measuring their structural features, which can impact the likelihood of ligand docking [22]. For example, a binding pocket must be accessible to a ligand, and the pocket must be large enough to accommodate the ligand. Protein structures used for analysis in ICM-Pro are obtained from the Protein Data Bank (PDB). Figure 1 shows examples of the analysis of ICM-Pro on protein structures with pockets without (Figure 1A) and with docked ligands (Figure 1B). 

**Figure 2:**
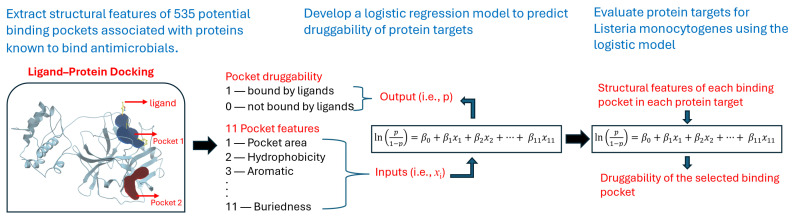
The framework of our druggability analysis approach for protein targets of *L. monocytogenes*.

The framework of our druggability analysis approach for protein targets of *L. monocytogenes* is illustrated in Figure 2. A reference set of 46 proteins was carefully chosen to encompass both pockets with and without known binding ligands. While more protein targets can be chosen, this work is mainly focused on proteins that are targets of inhibitors and FDA-approved antimicrobials (mainly against *L. monocytogenes*) and that have known crystal structures. Given the protein structures, a pocket searching program (i.e., ICM Pocket Finder) determines the locations of the pockets and extracts their structural features (e.g., aromatic, Figure 2). The structural features for the reference proteins with known binding antimicrobials were evaluated to determine whether there were differences in structural features between pockets with and without docked ligands. Key structural features were then determined and used as the input to develop a logistic regression (LR) model to predict the likelihood of ligand binding in a pocket. The pocket LR model was then used to assess the likelihood of ligand binding to pockets in a target set of proteins found in the literature for *L. monocytogenes*.

### 2.1. Materials

#### 2.1.1. Reference Protein Selection to Build the Druggability Analysis Model

The approach developed in this work started with a set of reference proteins that are considered druggable. The reference set was generated using three criteria: (1) the protein structure is available in the PDB so that it can be analyzed in the ligand–protein docking program Molsolf ICM-Pro; (2) the source organism for the protein is bacterial (preferably *L. monocytogenes*) since the work is focused on *L. monocytogenes*; and (3) the protein is druggable (either confirmed in the literature or because the PDB structure contains a ligand) so that differences between pockets with and without ligands can be evaluated. Using these criteria, a reference set of 46 proteins that were bound by ligands, including known inhibitors and FDA-approved drugs, was established. The reference proteins were selected in multiple steps. In the first step, a literature review of druggable proteins was conducted, and 10 proteins were identified for which structures with ligands were also available in the PDB. In the second step, all structures in the PDB for proteins associated with *L. monocytogenes* were reviewed, and 20 structures with docked ligands were selected. Lastly, an additional 16 structures with docked ligands were identified using the “Find Similar Assemblies” feature in the PDB. The selected reference proteins are summarized in Table A1 in Appendix A.

#### 2.1.2. Physical Characterization of Protein Structures

Molsoft ICM-Pro is a modeling software program with tools for molecular modeling and docking [23]. It has multiple features, including ICM Pocket Finder, which identifies potential ligand binding sites, i.e., pockets in protein structures, and calculates the physical properties of the binding sites [24]. Pocket Finder determines the pockets from a three-dimensional protein structure using a predicting algorithm that has been validated using data from the PDB [22]. As part of this validation, it was also shown that prediction results are not significantly impacted by differences between the apo and bound forms of pockets [22]. Given a protein structure, ICM Pocket Finder returns a list of pockets and results for the 13 structural features that represent the physical characteristics of each pocket. The structural features are described in Table 1. Protein structure analysis was performed using ICM-Pro version 3.9-3a for Linux.

ICM Pocket Finder’s analysis of a protein uses three-dimensional structures from the PDB database [26]. The PDB stores coordinate files for each protein, listing the atoms in the protein and their locations relative to each other in the molecule [27]. The structures, determined using methods such as X-ray crystallography, NMR spectroscopy, and cryo-electron microscopy, are submitted to the PDB by structural biologists [27]. Each structure is given a unique identifier and includes a header that gives basic information about the protein. If the structure of a protein is available in the Protein Data Bank, the PDB identifier can be searched directly in ICM-Pro, and the structure can be loaded and analyzed with ICM Pocket Finder.

#### 2.1.3. Target Protein Selection for Druggability Analysis

A primary objective of this work is to perform a druggability analysis of high-potential drug targets to control *L. monocytogenes.* Prior studies have identified key genes and proteins involved in the stress response [14,28], virulence [8,29], and antibiotic resistance [10] of *L. monocytogenes*, as well as the interaction among all three processes [30]. Twenty-three key proteins were thus selected from these studies as high-potential targets for druggability analysis. These proteins were selected because they play an important role in the stress response, antibiotic resistance, and virulence of *L. monocytogenes,* and their structures are available in the PDB. The target proteins are listed in Table A2 in Appendix B. 

### 2.2. Methods

The structural features were compared between the pockets with known binding ligands and the pockets without a ligand. All statistical calculations described in this section were performed using the free statistical software package RStudio, version 2023.12.1 Build 402, created by Posit Software, PBC (Boston, MA, USA) [31]. The logistic regression model was generated in R using the “glm” command from the package “base” version 4.3.2. The McFadden R^2^ was calculated in R using the “pR2” command, which is part of the package “pscl” version 1.5.9. The commands used in R for additional calculations are described in each section.

#### 2.2.1. Statistical Analysis of Pocket Data for Reference Proteins

Summary statistics were calculated for each structural feature of the pocket data for the reference proteins to determine whether there were statistically significant differences between pockets with and without ligand docking. These statistics serve as justification for selecting pocket features as the inputs of the logistic regression model. First, a Shapiro–Wilk test was performed to determine whether the data for each of the pocket parameters were normally distributed. For normally distributed data, a two-sided *t*-test was performed with a 95% confidence interval and the hypothesis that there is no difference between the means of the parameter data with and without ligands. For non-normally distributed data, a Mann–Whitney test was performed to determine whether there is a difference in the parameter data with and without the ligand. The Shapiro–Wilk test was performed in R using the “shapiro.test” command, the *t*-test was performed in R using the “*t*-test” command, and the Mann–Whitney test was performed in R using the “wilcox.test” command. Each of these commands is part of the package “stats” version 4.3.2.

#### 2.2.2. Preparation of the Reference Data Set 

Prior to generating the logistic regression model, the pocket features were normalized to the range of 0 to 1. This allows a direct comparison of the magnitudes of the logistic regression coefficients to determine the relative impact of each parameter on the predicted result. The pocket feature data for the reference proteins with known binding ligands and target proteins for *L. monocytogenes* were combined into one data set for normalization to ensure they were on the same scale. The structural feature data returned by Pocket Finder for the reference and target proteins are included in the supplementary information File S1.

The full normalized reference data set was evaluated in R for pair-wise correlations. This was carried out using the “Find Correlation” command from the package “caret” version 6.0–94. The volume, area, and radius features were determined to be correlated to each other based on a correlation cutoff value of 0.9. Since collinearity can affect the variances, signs, and magnitudes of logistic regression coefficient estimates [32], the volume and radius were not included in the logistic regression model. While any one of the three correlated features could have been kept, it was determined to keep the area (removing volume and radius) by generating a model for all three cases and selecting the one with the highest McFadden R^2^ value. The R^2^ values for the model with volume (with area and radius removed), area (with volume and radius removed), and radius (with volume and area removed) were 0.4454, 0.4507, and 0.4456, respectively. As a result, 11 of the 13 features listed in Table 1 were used for the model. An additional parameter called ligDock was created and added to the reference data to represent the druggability of each protein pocket. It was assigned a value of 0 for pockets without docked ligands and a value of 1 for pockets with docked ligands. It was used as the output of the logistic regression model. 

#### 2.2.3. Logistic Regression

Logistic regression (LR) can be used with a binomial dependent variable and multiple independent variables. The LR model has the following form:

(1) $$\ln\left(\frac{p}{1-p}\right)=\beta_{0}+\beta_{1}x_{1}+\beta_{2}x_{2}+\cdots +\beta_{11}x_{11}$$

where p is the probability of the binding pocket in the protein target being bound by a ligand, $x_{i}$ denotes the independent pocket feature, and $\beta_{i}$ denotes the regression coefficients [33]. For this work, the output variable *p* is represented by ligDock, which has a value of 1 if a pocket contains a ligand and 0 if it does not contain a ligand. A protein that has a pocket that contains a ligand (ligDock = 1) is druggable, whereas a protein that does not contain any pockets with ligands (ligDock = 0) is considered to be non-druggable. The independent variables *x*_1_, *x*_2_, …, *x*_11_ are pocket characteristics calculated in ICM-Pro (representing all the features in Table 1 except volume and radius). Generating the LR model estimates the regression coefficients *β*_0_, *β*_1_, … *β*_11_ for each of the independent variables. These represent the extent to which each of the features impacts the dependent variable, i.e., druggability. 

Estimating the coefficients for the LR model requires a training data set. For this analysis, the reference data were randomly split into two data sets: one to train the model, i.e., to estimate the regression coefficients, and one to test the predictability of the model. The reference data set consisted of 46 proteins that resulted in 535 pockets, of which 138 contained docked ligands, and 397 did not contain docked ligands. Approximately 50% of the reference data were used to train the model. As a result, the training data set had 268 pockets, of which 61 contained docked ligands. The test data set had 267 pockets, of which 77 contained docked ligands. 

#### 2.2.4. Analysis of the Target Data Set

The program ICM Pocket Finder was also used to generate pocket data for the target proteins identified from the literature for *L. monocytogenes*. The 23 target proteins contained 238 pockets. The target feature data were normalized together with the reference data prior to analysis. The LR model developed for the reference protein set was used to predict the chances of ligand docking in the pockets for each of the 23 target proteins for *L. monocytogenes*.

## 3. Results

### 3.1. Summary Statistics of Pocket Data for Reference Proteins

**Figure 3:**
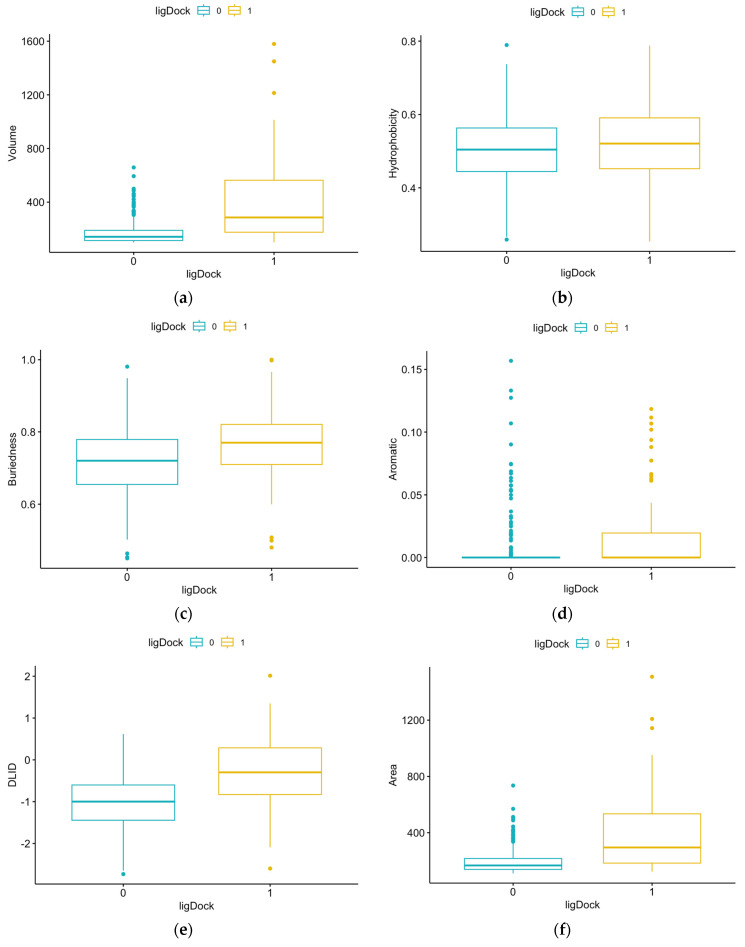

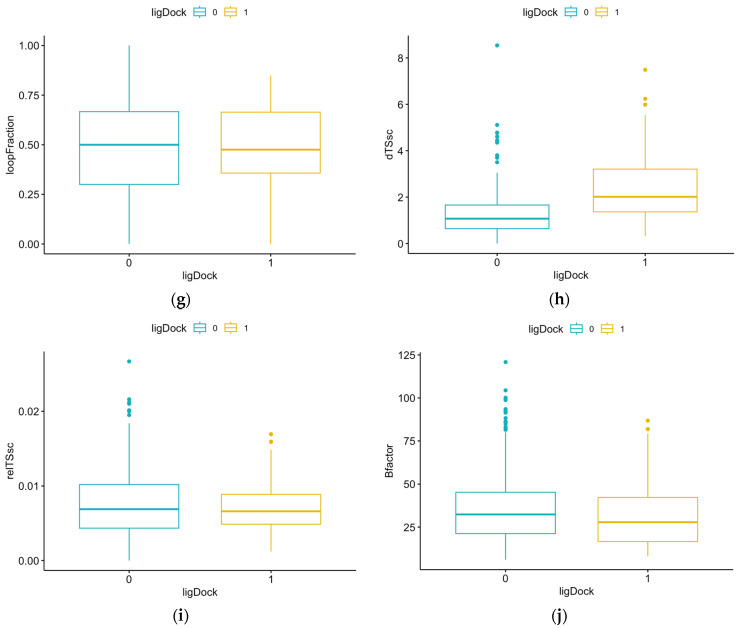

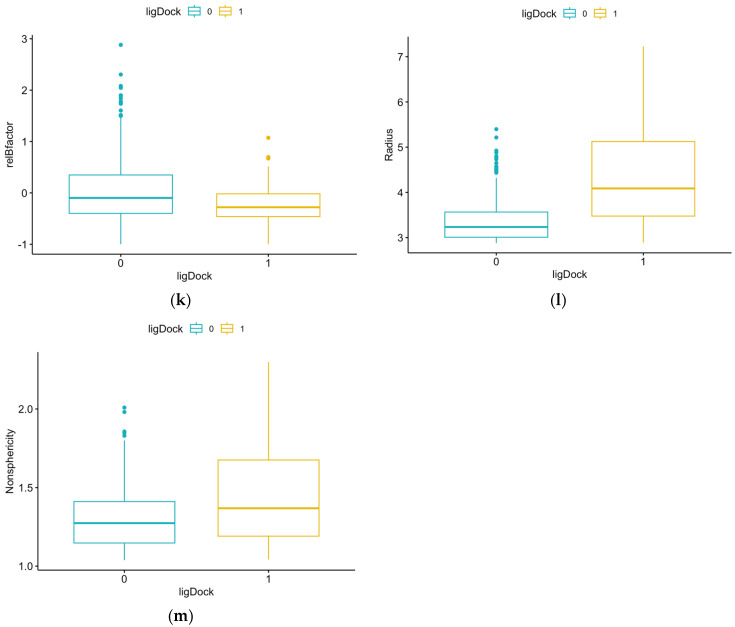
Comparison of results for each structural feature for pockets with and without ligands: (**a**) volume, *p*-value = 2.2 × 10^−16^ (Mann–Whitney); (**b**) hydrophobicity, *p*-value = 0.0417 (*t*-test); (**c**) buriedness, *p*-value = 1.734 × 10^−7^ (*t*-test); (**d**) aromatic, *p*-value = 1.309 × 10^−12^ (Mann–Whitney); (**e**) DLID, *p*-value = 2.2 × 10^−16^ (*t*-test); (**f**) area, *p*-value = 2.2 × 10^−16^ (Mann–Whitney); (**g**) loopFraction, *p*-value = 0.6116 (Mann–Whitney); (**h**) dTSsc, *p*-value = 2.2 × 10^−16^ (Mann–Whitney); (**i**) relTSsc, *p*-value = 0.5352 (Mann–Whitney); (**j**) Bfactor, *p*-value = 0.001695 (Mann–Whitney); (**k**) relBfactor, *p*-value = 1.906 × 10^−5^ (Mann–Whitney); (**l**) radius, *p*-value = 2.2 × 10^−16^ (Mann–Whitney); (**m**) nonsphericity, *p*-value = 8 × 10^−7^ (Mann–Whitney).

Summary statistics for each of the pocket features for the reference proteins were calculated. Per the Shapiro–Wilk test, the data were normally distributed for hydrophobicity, buriedness, and DLID, and the data for all other parameters were not normally distributed. The two-sided *t*-test was performed for normally distributed data, and the Mann–Whitney test was performed for non-normally distributed data. The data sets were determined to be statistically different for 11 of the 13 features (excluding loopFraction and relTSsc) with a significant *p*-value. Figure 3 shows the box plot comparisons for each of the features using the un-normalized data and the *p*-values for the statistical tests (i.e., two-sided *t*-test or Mann–Whitney test).

### 3.2. Logistic Regression Model Results 

Table 2 summarizes the LR model coefficients calculated using the training data. The *p*-values are significant for 5 out of the 12 coefficients. The McFadden R^2^, which is a measure of the resolution of the model between predicted states [34], is 0.45 for the full model. A reduced version of the model was calculated using only the independent variables for which the *p*-values were significant. This resulted in a model for which the McFadden R^2^ was 0.41. A second reduced version of the model was calculated with loopFraction and relTSsc variables excluded because there was not a statistically significant difference in the means of these parameters with and without docking (see Figure 2). The McFadden R^2^ for this version of the model was 0.43. Since the R^2^ was higher, the full model was used for all subsequent results.

**Figure 4:**
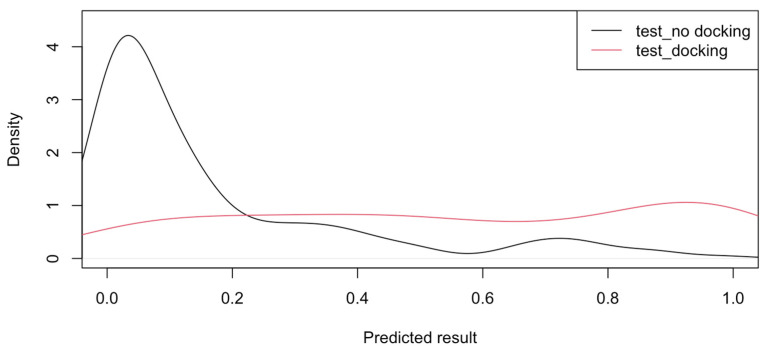
Density plot for predicted results for test data.

The LR model was tested by using it to predict the results for pockets for the reference testing data. Figure 4 shows the density plot for model predictions for the test data. The predicted results for pockets without a ligand are clustered near zero, as expected. However, the predicted results for pockets with ligands are spread out across a wider range of values. This indicates that the pocket characteristics are more consistent, and the model can more accurately identify pockets with no ligands.

The LR model generated by the “glm” command in R outputs a continuous result that represents the probability of a binary outcome of 1 or 0. To classify the predicted results as 1 or 0, a cutoff value is selected such that values greater than or equal to the cutoff value are classified as 1, and values less than the cutoff value are classified as 0. The optimal cutoff value was determined using the model to predict the results for the test data with a range of cutoff values. Based on the results shown in Table 3, a cutoff value of 0.50 was selected because it resulted in the highest accuracy and the highest specificity for the model. This cutoff value ensures the highest chance of correctly predicting “docking” for a pocket with docking but also increases the possibility of incorrectly identifying “no docking” as “docking”.

Using a cutoff value of 0.5, the LR model was used to predict the results for the test data. The results are shown in Table 4.

**Figure 5:**
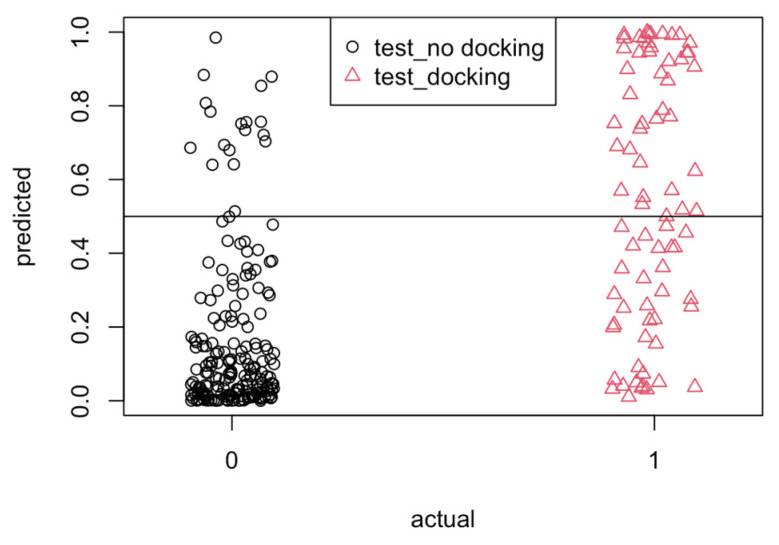
Confusion matrix of predicted results for test data (horizontal line y = 0.5 indicates cutoff value).

The classification of predicted results for the test data is shown graphically in Figure 5, referred to as the confusion matrix. With a cutoff value of 0.5, the overall prediction accuracy for the model evaluating the testing data set was 80%. For actual values of 0, i.e., “no docking”, 91% of the values were correctly predicted, i.e., below the horizontal line at y = 0.5. For actual values of 1, i.e., “docking”, 55% of the values were correctly predicted, i.e., above the horizontal line at y = 0.5.

### 3.3. Predicted Results for Target Proteins

The chance of docking was calculated for target proteins. For the 23 target proteins, 28 of the 238 pockets had predicted results of ligDock = 1, i.e., “docking”. These represent 9 of the 23 target proteins. If at least one pocket for a protein demonstrates druggability (i.e., with an output of 1 from the LR model), the protein is considered a druggable target. Table 5 lists the target proteins that are predicted to be druggable by the LR model, with the PDB ID and the name of the protein (within the parenthesis).

## 4. Discussion

### 4.1. Logistic Regression Model

The primary purpose of this work was to develop an approach to assessing the druggability of proteins. A reference set of proteins considered to be druggable was created by performing a literature search for druggable proteins and searching the PDB for all *L. monocytogenes* protein structures with ligands and similar assemblies. The reference set of 46 proteins consisting of 535 pockets was analyzed. The summary statistics for the pocket features confirm that there are differences in the results between pockets with and without docked ligands. This supports the premise of this work, i.e., that the pocket features of proteins can be used to assess their likelihood for ligand docking, which is an indicator of druggability.

The pocket data were normalized to allow a direct comparison of the coefficients that have the most impact on the predicted result based on magnitude. The sign of a coefficient also indicates whether the corresponding parameter is directly or inversely correlated to the likelihood of docking, i.e., druggability. In general, the coefficients agree with expectations. For example, DLID is positive with the largest magnitude. This parameter was previously developed to predict ligand docking, with larger values being better [20]. Buriedness and area also have larger magnitudes and signs that correspond with the expected impact. The buriedness parameter is the ratio of the pocket surface area covered by its shell to the total pocket surface, where 1.0 corresponds to completely buried. Therefore, it is expected that this would be inversely correlated to ligand docking since lower values correspond to the pocket being more accessible to ligands. Larger areas correspond to larger pockets, which minimize sizing constraints and would be expected to make the pockets more favorable for ligand docking. The main feature that contradicts expected behavior is hydrophobicity. It was expected that pockets with higher percentages of hydrophobic surface areas would be more favorable for ligand docking [20]; however, the LR model indicates that this parameter is inversely correlated to docking. A potential explanation for this is that the arrangement of hydrophobic groups in the binding site is also important, not just the overall hydrophobicity [20]. This logic model will be further improved by incorporating the information on local residues around the binding pockets. This is an interesting direction for future research. 

The density plot in Figure 4 shows that the model better predicts “no docking”. This is confirmed by the accuracy (80%), sensitivity (83%), and positive prediction (95%) results in Table 3 for the test data. For “docking”, the model is less accurate, as indicated by the specificity (70%) and negative prediction (55%) results in Table 3. This likely means that it is not necessary for all the features to be aligned for docking to occur. For example, based on the regression coefficients, high values of DLID, area, relTSsc, and Bfactor would contribute to a predicted result closer to 1. But the fact that the test results for docking are spread out, as shown in Figure 4, indicates that high values are not required for all these features for docking to be feasible. The selected cutoff value for the model was based on achieving the highest rate of true “docking” predictions for the test data. The model should be used with caution, as the likelihood of a false “docking” prediction is 17%, and that of a false “no docking” prediction is 30%. It is suggested that the model be used in conjunction with other tools, such as sequence-based or structure-based prediction, to evaluate potential protein targets.

### 4.2. Druggability Assessment of Target Proteins

The LR model was used to assess the druggability of a set of target proteins. These proteins were previously identified as key proteins involved in the stress response, virulence, and antibiotic resistance of *L. monocytogenes* and represent high-potential targets to better control the pathogen. As shown in Table 5, 9 of the 23 target proteins are identified as having a high likelihood of ligand docking and are therefore considered to be druggable. These are GroEL, FlgK, FlgL, FlhB, FliG, FliH/FliI complex, InlA, MogR, and PrfA. The functions of each of these proteins are specified in Table A2. Of particular interest are the PrfA protein, which is a regulator of all virulence proteins that are important during the infection process [35], and the various motility processes, which have a role in biofilm formation and antibiotic resistance [10].

The ability to find effective drugs for the proteins identified by the model can be impacted by the following issues. The method determines the druggability of the protein target based upon the geometric properties of potential binding pockets in the protein structure. It has been shown that binding kinetics, such as the association rate and dissociation rate, can influence the affinity of the ligand to the target and impact factors such as onset of action and duration of response [36]. If there are non-critical proteins involved in other pathways or cellular functions that have similar binding pockets and more favorable binding kinetics, these could affect the specificity of ligand binding to the target proteins. These are issues that should be considered in the evaluation of specific ligands as potential drugs for side-effect evaluation. 

### 4.3. Limitations

There are some limitations of the approach developed in this paper. First, the model requires the protein structure for ICM-Pro to extract the pocket feature data. While protein structures are increasingly available from multiple sources, structures are not available for all proteins. In addition, the model calculates the features for “docking” based on pockets with docked ligands and for “no docking” based on pockets without docked ligands. For pockets that do not contain ligands, they are characterized as “no docking”. However, this may be due to the fact that these pockets are not as competitive in attracting the binding compounds when compared to the pockets with binding ligands. It is not known whether these pockets absolutely cannot support ligand docking or whether structures are just not available with docked ligands in those pockets. 

Since the results are based on the structural features of the reference set of proteins, increasing the size of the reference set by using more proteins from other organisms and additional structures as they become available could improve the model’s accuracy. Lastly, since structures were not available in the PDB for all the target proteins from *L. monocytogenes*, structures for the proteins from other bacterial organisms were used, and there could be differences in the structures from different organisms. The results from this work can be further refined once the crystal structures of those proteins are available. 

## 5. Conclusions

*L. monocytogenes* is ranked third among foodborne pathogens in causing death. It can survive environmental stress, escape antimicrobial treatment, and invade human bodies to cause sickness. It thus poses a significant challenge to the food industry. While extensive research has been conducted to identify key proteins that facilitate the growth, antibiotic resistance, and virulence of *L. monocytogenes*, little work has been carried out to further evaluate the druggability of those proteins. To address this gap, a logistic regression model has been generated and tested to predict the druggability of protein targets based on the structures of binding pockets of proteins with known ligands (e.g., antimicrobials) and crystal structures. The developed logistic regression model was then used to evaluate 23 key proteins for *L. monocytogenes*, and the following proteins are predicted to be druggable targets: GroEL, FlgK, FlgL, FlhB, FliG, FliH/FliI complex, InlA, MogR, and PrfA. These results serve as an initial step for further drug discovery to identify specific compounds that can dock to the druggable target proteins and for experimental work to confirm that they are effective targets.

## Figures and Tables

**Table 1:** Summary of the structural features returned by ICM Pocket Finder [25].

Parameter	Definition
Volume	Pocket volume
Hydrophobicity	Percentage of pocket surface in contact with hydrophobic protein residues (in the range of 0–1)
Buriedness	Ratio of pocket surface area covered by its shell to total pocket surface area (1.0 means completely buried)
Aromatic	Fraction of pocket formed by aromatic side chains (higher is better)
DLID	Drug-like density score measuring bindability of proteins (slightly negative and above 0 are considered “druggable”)
Area	Pocket area
LoopFraction	Fraction of pocket formed by residues from loops (lower is better)
dTSsc	Estimate of entropic penalty associated with flexible side chains forming parts of the pocket
relTSsc	Same as dTSsc but relative to pocket volume (lower is better)
Bfactor	Average b-factor of pocket-forming atoms (lower is better)
relBfactor	Normalized deviation of pocket b-factor from the average over the protein (lower is better)
Radius	Radius of an ideal spherical cavity with the same volume as the pocket
Nonsphericity	Ratio of pocket area to ideal spherical cavity area (1.0 means completely spherical)

**Table 2:** Logistic regression model coefficients.

Variable (*x_i_*)	Coefficient (*β_i_)*	Std. Error	z Value	Pr (>|z|)
(Intercept)	3.9724	1.8218	2.180	0.029277
Hydrophobicity	−15.0826	4.2276	−3.568	0.000360
Aromatic	−1.6691	1.8166	−0.919	0.358196
Buriedness	−14.8371	5.5163	−2.690	0.007152
DLID	31.8352	10.1678	3.131	0.001742
Area	15.0366	12.9474	1.161	0.245494
loopFraction	−1.8166	1.1914	−1.525	0.127343
dTSsc	−7.0218	5.4238	−1.295	0.195450
relTSsc	5.9622	3.8447	1.551	0.120960
Bfactor	0.6733	3.4303	0.196	0.844396
relBfactor	−10.5689	3.0309	−3.487	0.000488
Nonsphericity	−9.0507	4.0022	−2.261	0.023732

**Table 3:** Cutoff value results for test data.

Cutoff Value	Accuracy ^1^	Sensitivity ^2^	Specificity ^3^	Positive Pred. Rate ^4^	Negative Pred. Rate ^5^
0.60	0.7790	0.8047	0.6731	0.9105	0.4545
0.50	0.8015	0.8309	0.7000	0.9503	0.5455
0.40	0.8015	0.8586	0.6579	0.8632	0.6494
0.30	0.7715	0.8644	0.5889	0.8053	0.6883
0.20	0.7491	0.9020	0.5439	0.7263	0.8052

^1^ Accuracy is the rate of correct predictions for “no docking” and “docking”. ^2^ Sensitivity is the rate of correctly predicting “no docking” for a pocket with “no docking”. ^3^ Specificity is the rate of correctly predicting “docking” for a pocket with “docking”. ^4^ The positive prediction rate is the rate of correct “no docking” predictions. ^5^ The negative prediction rate is the rate of correct “docking” predictions.

**Table 4:** Predicted results for test data (267 samples).

Prediction	Reference	
	0	1
0	172	18
1	35	42

**Table 5:** Predicted results for target proteins.

ligDock = 0	ligDock = 1 (Druggable)
1I5N (CheA) 1XEU (InlC) 2J70 (RsBU) 2PLC (PlcA) 2WQV (InlB) 2ZVY (MotB) 3FDQ (FlaA) 3MIX (FlhA) 4NL2 (Hfq) 5H5T (FliD) 6F2D (Flip) 7X1K (DegU) 8CQM (PlcB)	1O6V (InlA) 1XCK (GroEL) 3B0Z (FlhB) 4UT1 (FlgK) 5B0O (FliH/FliI complex) 5LEJ (PrfA) 5ZIY (FlgL) 7X9S (MogR) 8UMD (FliG)

## Data Availability

Data are available in the supplementary material.

## Supplementary Materials

The following supporting information can be downloaded at: https://www.mdpi.com/article/10.3390/microorganisms12061073/s1, File S1: The structural feature data returned by Pocket Finder for the reference and target proteins.

## Appendix A

**Table A1:** Summary of reference proteins.

PDB Entry	Protein	Organism	Ligand	Source
1C14	Ndah	*E. coli*	Triclosan	[37]
1E9X	Cyp51	*M. tuberculosis*	4-Phenylimidazole	PDB search for similar assemblies
1EA1	Cyp51	*M. tuberculosis*	Fluconazole	[37]
1I2Z	Ndah	*E. coli*	Imidazole	PDB search for similar assemblies
1KIJ	GyrB	*T. thermophilus*	Novobiocin	[37]
1KZN	GyrB	*E. coli*	Clorobiocin	[37]
1QMF	Pbp2X	*S. pneumoniae*	Carboxylic acid	[37]
1TZ6	AirK	*S. enterica*	Aminoimidazole riboside	PDB search for similar assemblies
2Q3J	Cpfc	*B. subtilis*	N-methyl mesoporphyrin	PDB search for similar assemblies
3DA1	Gpdh	*B. halodurans*	Flavin-adenine dinucleotide	[38]
3SYN	FlhF	*B. subtilis*	Guanosine-5′-diphosphate	PDB search for similar assemblies
3TFC	AroA	*L. monocytogenes*	Phosphoenolpyruvate	PDB search for *Lm protein complexes*
3TNL	SkdH	*L. monocytogenes*	Shikimate and NAD	[38]
3TOZ	SkdH	*L. monocytogenes*	Nicotinamide-adenine-dinucleotide	PDB search for *Lm protein complexes*
3U9E	Bup/Acp	*L. monocytogenes*	Coenzyme A	PDB search for *Lm protein complexes*
3UF6	Bup/Acp	*L. monocytogenes*	3′-Dephosphocoenzyme A	PDB search for *Lm protein complexes*
3ZG8	Pbp4	*L. monocytogenes*	Ampicillin	PDB search for *Lm protein complexes*
3ZG9	Pbp4	*L. monocytogenes*	Cefuroxime	PDB search for *Lm protein complexes*
3ZGA	Pbp4	*L. monocytogenes*	Carbenicillin	PDB search for *Lm protein complexes*
4INJ	MccF	*L. monocytogenes*	Methyl sulfamate	PDB search for *Lm protein complexes*
4JRO	FabG	*L. monocytogenes*	Nicotinamide-adenine-dinucleotide phosphate	PDB search for *Lm protein complexes*
4RWW	PstA	*L. monocytogenes*	Cyclic-di-AMP	PDB search for *Lm protein complexes*
4S1B	PgpH	*L. monocytogenes*	Cyclic-di-AMP	PDB search for *Lm protein complexes*
5B0O	FliH/FliI	*S. enterica*	Adenosine-5’-diphosphate	PDB search for similar assemblies
5DHP	NadK1	*L. monocytogenes*	Novel inhibitor	PDB search for *Lm protein complexes*
5F1R	PrfA	*L. monocytogenes*	Ring-fused 2-pyridone (C10)	PDB search for *Lm protein complexes*
5F7V	Lmo0181	*L. monocytogenes*	Cycloalternan	PDB search for *Lm protein complexes*
5TED	QuiR	*L. monocytogenes*	Shikimate	PDB search for *Lm protein complexes*
5UPX	ImpDH	*L. monocytogenes*	Xanthosine monophosphate	PDB search for *Lm protein complexes*
5VJD	FbaA	*E. coli*	Dihydroxyacetonephosphate	[38]
5ZQB	PbpD2	*L. monocytogenes*	Penicillin G	PDB search for *Lm protein complexes*
5ZQC	PbpD2	*L. monocytogenes*	Ampicillin	PDB search for *Lm protein complexes*
5ZQD	PbpD2	*L. monocytogenes*	Cefotaxime	PDB search for *Lm protein complexes*
5ZQE	PbpD2	*L. monocytogenes*	Cefuroxime	PDB search for *Lm protein complexes*
6C5N	IlvC	*S. aureus*	Hydroxymate inhibitor 1	[38]
6C8Q	NadE	*E. faecalis*	Nicotinamide-adenine-dinucleotide	PDB search for similar assemblies
6FXJ	ChdC	*L. monocytogenes*	Iron coproporphyrin III	PDB search for *Lm protein complexes*
6HVL	CdaA	*L. monocytogenes*	Cyclic-di-AMP and AMP	PDB search for *Lm protein complexes*
6O6N	FasR	*M. tuberculosis*	Arachinoyl-Coenzyme A	PDB search for similar assemblies
6XXY	LeuB	*H. influenzae*	O-isobutenyl oxalylhydroxamate	[38]
7NNV	ArgF	*M. tuberculosis*	Carbamoyl phosphate	PDB search for similar assemblies
7XMD	Cytbo3	*E. coli*	Allosteric inhibitor N4	PDB search for similar assemblies
8EBC	ImpDH	*L. monocytogenes*	Inosinic acid	PDB search for *Lm protein complexes*
8H62	InlA	*L. monocytogenes*	E-cadherin EC12	PDB search for *Lm protein complexes*
8UVZ	DhfR	*B. subtilis*	Nicotinamide-adenine-dinucleotide and folate	PDB search for similar assemblies
8VDA	FabH	*B. subtilis*	Coenzyme A	PDB search for similar assemblies

## Appendix B

**Table A2:** Summary of the target proteins.

PDB Entry	Protein	Function	Source
1AOD	PlcA	Plays a role in transmembrane signaling.	[35]
1I5N	CheA	A chemotaxis protein involved in the transmission of sensory signals from chemoreceptors to flagellar motors.	[39]
1O6V	InlA	A surface protein that mediates the attachment to and invasion of host cells.	[35]
1XCK	GroeL	Prevents misfolding and promotes refolding and proper assembly of unfolded polypeptides generated under stress conditions.	[40]
1XEU	Inlc	Stimulates the formation of membrane protrusions that mediate the intercellular spread of *L. monocytogenes.*	[35]
2J70	RsbU	Acid, antibiotic, cold, ethanol, heat, osmotic and nutritional stress responses require rsbU to activate sigB.	[41]
2PLC	PlcA	Plays a role in transmembrane signaling.	[35]
2WQV	InlB	A surface protein that mediates attachment to and invasion of host cells.	[35]
2ZVY	MotB	A motility protein.	[42]
3B0Z	FlhB	A motility protein; a membrane protein responsible for substrate specificity, switching from rod/hook-type export to filament-type export.	[30]
3FDQ	FlaA	A motility protein; a flagellin protein that polymerizes to form the filaments of bacterial flagella.	[30]
3MIX	FlhA	A motility protein; a membrane protein involved in the flagellar export apparatus.	[30]
4NL2	Hfq	A regulatory factor involved in the stress response and virulence.	[43]
4UT1	FlgK	A motility protein; a flagellar hook protein; acts as a hook filament junction protein with flgL to join the flagellar filament to the hook.	[30]
5B0O	FliH/FliI	Involved in type III protein export during flagellum assembly.	[30,44]
5H5T	FliD	A motility protein; a flagellar hook protein; required for morphogenesis and for the elongation of the flagellar filament.	[30]
5LEJ	PrfA	A transcriptional activator of virulence genes.	[35]
5ZIY	FlgL	A motility protein; Lmo0706; acts as a hook filament junction protein with FlgK to join the flagellar filament to the hook.	[30]
6F2D	FliP	A motility protein; forms the core of the central channel in the flagella export apparatus with proteins fliQ and fliR.	[30]
7X1K	DegU	A stress response regulator.	[45]
7X9S	MogR	A transcriptional repressor required for virulence.	[46]
8CQM	PlcB	Plays a role in transmembrane signaling.	[35]
8UMD	FliG	One of three proteins involved in switching the direction of the flagellar rotation.	[30]
